# Revealing eRNA interactions: TF dependency and convergent cooperativity

**DOI:** 10.21203/rs.3.rs-2592357/v1

**Published:** 2023-02-28

**Authors:** Seungha Alisa Lee, Katla Kristjánsdóttir, Hojoong Kwak

**Affiliations:** Cornell University; Cornell University; Cornell University

## Abstract

Enhancer RNAs (eRNAs) are non-coding RNAs produced from transcriptional enhancers that are highly correlated with their activities. Using capped nascent RNA sequencing (PRO-cap) dataset in human lymphoblastoid cell lines across individuals, we identified inter-individual variation of expression in over 80 thousand transcribed transcriptional regulatory elements (tTREs), in both enhancers and promoters. Co-expression analysis of eRNAs from tTREs across individuals revealed how enhancers interact with each other and with promoters. Mid-to-long range interactions showed distance-dependent decay, which was modified by TF occupancy. In particular, we found a class of ‘bivalent’ TFs, including Cohesin, which both facilitates and insulates the interaction between enhancers and/or promoters depending on the topology. In short ranges, we observed strand specific interactions between nearby eRNAs in both convergent or divergent orientations. Our finding supports a cooperative convergent eRNA model, which is compatible with eRNA remodeling neighboring enhancers rather than interfering with each other. Therefore, our approach to infer functional interactions from co-expression analyses provided novel insights into the principles of enhancer interactions depending on the distance, orientation, and the binding landscapes of TFs.

## Introduction

Regulation of transcription is achieved mainly through the binding of transcription factors (TFs) at transcription regulatory elements (TREs), such as promoters and enhancers^1–4^. Genes are expressed from promoters, where regulatory signals are integrated from proximal and distal enhancers to determine the amount of RNA product. Enhancers act as distant regulatory elements for promoters through specific TF binding, while at the same time produce bidirectional enhancer RNAs (eRNAs). Regulatory networks by enhancers are key to most cellular processes, including development, cell-type differentiation, and stress response, whereas their misregulation could cause disease. Numerous studies revealed that a large majority of disease associated genetic variations affect TREs^5–14^. Understanding TRE networks requires knowledge of how TREs interact with one another, and the mechanisms through which this regulation is achieved.

The bidirectional production of RNA from TREs, including both enhancers and promoters, is one of the most important hallmarks of regulatory activity in vertebrates, as transcribed enhancers are most active^1–4^. Consequently, relying on RNA products to identify TREs emerged as an efficient approach. For instance, the FANTOM5 consortium used Cap Analysis of Gene Expression (CAGE) to create an atlas of enhancer activity across numerous cell types and tissues^6^. While CAGE is a simple and powerful method to quantify transcription initiation at genes, it is less well suited for the quantification of transcriptional activity at enhancers, which produce particularly unstable eRNAs. Sequencing methods that capture nascent RNA, such as global Precision nuclear Run-On sequencing with 5´-capped (m^7^G) RNA enrichment (GRO-cap or PRO-cap), measure transcriptional activity directly and are, therefore, better suited to the quantification of TRE activity^2,15^.

After identifying and quantifying active TREs, which TREs control gene expression and how they do so becomes an eminent question. Systematic analysis of expression variation can reveal these targets and mechanisms. Co-expression network uses variation in expression across different samples to elucidate regulatory circuits^16^. By coupling co-expression analysis to TF binding profiles, the mechanisms of the regulatory circuits can be characterized. While physical interaction between TREs, charted through chromatin conformation assays^18–21^, remains the gold standard for mapping chromatin interaction networks, such interactions are not always readily detectable. Functional interactions identified based on TRE co-expression can, therefore, complement physical interaction maps to fill gaps in the regulatory network.

This study leverages the variation in transcription initiation at transcribed TREs (tTREs), majority of which are eRNAs^19^. We investigate the interactions between tTREs measured by PRO-cap in lymphoblastoid cell lines (LCLs) from ~ 70 individuals. We use co-variation between tTREs as an indicator of functional interactions between sites and explore how those interactions are globally affected by various transcription factors. We identified thousands of putative interactions and found global signatures of either facilitation or inhibition of interactions for multiple TFs. We also explored interactions between the different strands of neighboring tTREs and found evidence of cooperativity at sites with converging polymerases, discovering new rules of eRNA interactions and their potential roles.

## Results

### Co-expression of tTREs as an indicator of functional interaction

**Figure 1:**
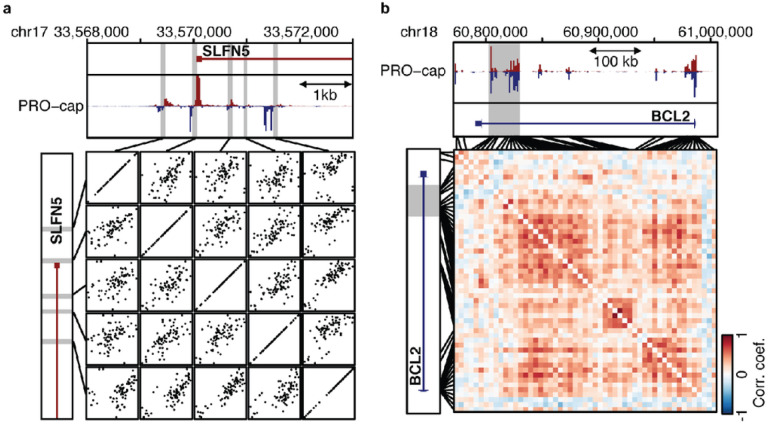
Covariation of tTRE transcription as an indicator of functional interactions. (**a**) High-resolution example of the co-expression analysis strategy at the SLFN5 locus. The PRO-cap signal at each variably expressed tTRE (proximal enhancer or promoter) is correlated with the PRO-cap signal of every other variably expressed tTRE in the vicinity, across LCL samples. (**b**) Co-expression across a larger region at the BCL2 locus reveals putative functional interactions both within and between tTRE clusters. Heatmap represents correlation coefficients for correlations between tTRE pairs across LCL samples.

While approaches such as physical interaction maps have defined and provided insight into the principles of cis-regulatory modules^18–21^, we leverage our previously published PRO-cap datasets from ~70 YRI LCLs to study functional interactions between tTREs using a tTRE co-expression analysis^17^. This dataset includes 87,826 tTREs, of which about 1/5 correspond to promoters and the remainder are defined as putative enhancers. These were identified based on bidirectional transcription of a pair of nascent RNAs within 300 bps of each other. Of these, about 40% are variably expressed across the individuals. As a measure of co-expression, we used Pearson’s correlation coefficients for linear regressions across individuals of the PRO-cap signals between pairs of variably transcribed tTREs in short range near SLFN5 gene promoter and in long range at BCL2 super-enhancer locus (Figs. 1a, 1b). The example at the BCL2 locus illustrates how this analysis allows us to visualize which TREs are likely to have functional interactions, both within clusters of tTREs and between such clusters.

**Figure 2:**
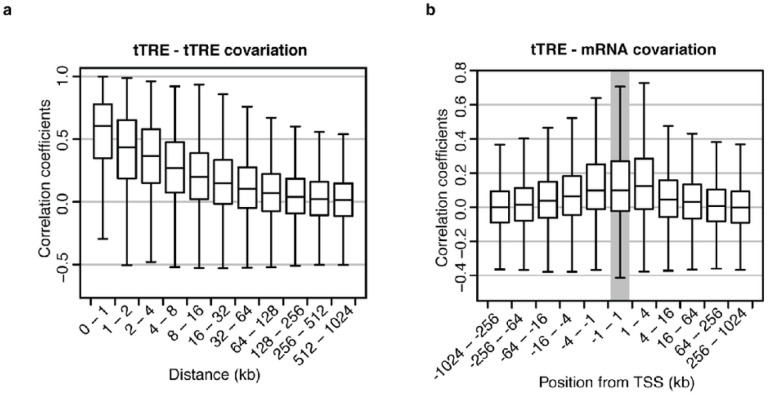
TF binding sites at or between tTREs are associated with differences in tTRE co-expression. (**a**) Co-expression of tTREs decreases with increasing distance. tTRE pairs are binned according to the distance between them and the distribution of correlation coefficients in each bin is plotted as a boxplot. (**b**) Covariation between PRO-cap read-counts at tTREs and mRNA expression levels (RNA-seq^8^) varies according to both distance and orientation. Pairs were binned based on both the distance between the tTRE and the mRNA TSS, and the orientation of the pairing (TRE upstream: negative numbers, tTRE downstream: positive numbers). The distribution of correlation coefficients in each bin is plotted as a boxplot.

We explored whether the co-expression reveals the principles of functional interactions between tTREs, such as the rule of distance. When we bin the correlation coefficients based on the distance between tTREs, covariation of the PRO-cap levels decreases as the distance increases (Fig. 2a, **Supplementary Figs. 1a, 1b**). The distribution of the coefficients reaches background levels (comparable to inter-chromosomal interactions) by 1 Mb (**Supplementary Figs. 1c, 1d**), and the majority of the coefficients above background are for tTRE pairs within 200 kb (**Supplementary Fig. 1a**). This distance effect is unlikely to be bias from linkage disequilibrium (LD) of the genotypes at tTRE pairs, since the YRI population is known to have LDs smaller than 5 kb^22^ and a similar degree of distance decay is observed using only the tTRE pairs with independent genotypes (genotype correlation <0.05, **Supplementary Fig. 1e**).

We also explored how covariation between variably transcribed tTREs and mRNA levels varied based on the distance between the tTRE and gene promoters or mRNA TSSs (Fig. 2b, **Supplementary Figs. 1f, 1g**). The tTREs located upstream of the TSSs have a covariation pattern that gradually decreases upon distance. Interestingly, coefficient distribution to the downstream tTREs seem to drop faster with increasing distance. This is consistent with a recent report that intragenic enhancers are less activating for their host gene, and can even attenuate expression^23^.

### TF binding sites at or between tTREs are associated with differences in tTRE co-expression

Binding of transcription factors can modify interactions between TREs. Insulator proteins such as CTCF can disconnect the communication between two regions^24^ and transcriptional co-activators such as P300 can bridge TREs to their targets^25^. We tested these using published chromatin immunoprecipitation sequencing (ChIP-seq) datasets from ENCODE’s Factorbook repository^26^ in a representative LCL (GM12878) to determine the effect of TF binding on the global tTRE interaction trends.

**Figure 3:**
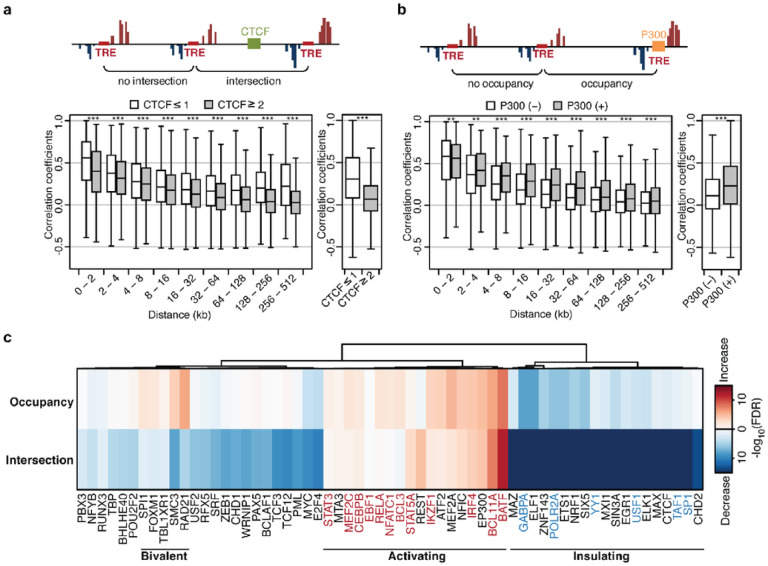
Transcription factor binding sites are associated with changes in tTRE co-expression. (**a**) Intersection by insulator decreases tTRE co-expression. Top: Diagram of CTCF bound to intersecting regions between two tTREs. Bottom: Distributions of correlation coefficients for tTRE pairs that are intersected by 2 or more CTCF sites and 1 or less, binned by distances (left) or total (right). *** indicates *P* < 1 × 10^−3^ by Wilcoxon rank sum test. (**b**) Occupancy by co-activator increases tTRE co-expression. Top: Diagram of P300 occupying a tTRE. Bottom: Correlation coefficients for tTRE pairs that are occupied by P300 or not occupied, binned by distances (left) or total (right). *** indicates *P* < 1 × 10^−3^, and ** indicates P < 1 × 10^−2^ by Wilcoxon rank sum test. (**c**) The effect of TF intersection and occupancy on tTRE interactions for 61 TFs. Heatmap indicates the −log_10_(FDR) for AUC between with and without the TF (Supplementary Figs 2b, 2c). The TFs are ordered by minimum variance hierarchical clustering.

To examine the insulating effect of CTCF on functional interactions, we separated tTRE pairs into “no intersection” and “intersection” categories based on the number of CTCF ChIP-seq peaks^27^ between them and plotted the distribution of correlation coefficients as a function of distance (Fig. 3a, **Supplementary Fig. 2a**). The presence of CTCF sites between tTREs reduced co-expression overall and within each distance bin. We also looked at the activating effect of P300 on co-expression, comparing tTRE pairs that are occupied by P300^26^ to ones where P300 is not bound. As expected, P300-bound tTRE pairs tend to have higher levels of co-expression than the unoccupied pairs (Fig. 3b, **Supplementary Fig. 2a**). These examples show that our co-expression analysis is able to detect the expected effects of TF binding, both when the TF is intersecting and when it is occupying the tTREs.

We expanded our analysis to all 60 TFs for which ChIP-seq data were available from the ENCODE repository^26^. To generate a metric of how TF binding modifies the distance-dependent decay of co-expression, we focused on tTRE interactions in the top 5^th^ percentile of the correlation coefficient distribution at each distance bin, and plotted the top 5^th^ percentile as a function of the distance. We then calculated the difference in the area under the curve (ΔAUC) between “occupancy”/”no occupancy” and “intersection”/”no intersection” tTRE pairs. We compared the ΔAUC to permuted background ΔAUC distributions to evaluate the significance of the difference (**Supplementary Figs. 2b, 2c**).

The TF analysis reveals three broad categories of TFs: insulating, activating, and bivalent (Fig. 3c, **Supplementary Fig. 2d**). Insulating TFs, such as CTCF, reduce tTRE co-expression both when intersecting and occupying tTREs. The opposite is true for activating TFs, such as P300. Interestingly, the activating category contains many TFs that are immune or B-cell specific (Fig. 3c, red), whereas the insulating category contains general factors associated with strong promoters (Fig. 3c, blue). Bivalent TFs, such as the cohesin subunits RAD21 and SMC3, enhance covariation when occupying the tTREs, but repress covariation when intersecting tTREs. Another bivalent factor, FOXM1, that controls cell cycle progression is also known to function as both repressor and activator depending on the chromatin context^28^.

### Strand-specific covariation at closely-spaced tTREs supports a cooperative model for convergent transcription

**Figure 4:**
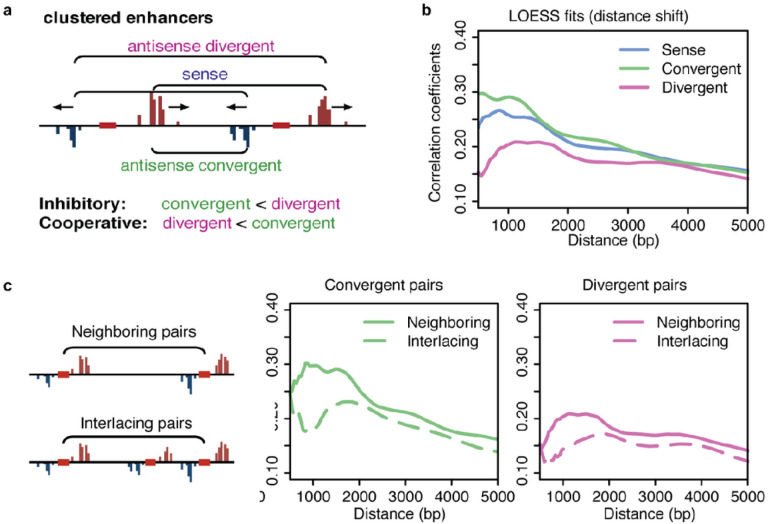
Covariation analysis for convergent and divergent strands of neighboring tTREs. (**a**) Diagram illustrates three strand comparisons for two neighboring tTREs. Sense comparisons (purple) are strands that are transcribed in the same direction, antisense comparisons are between strands that are transcribed in opposite directions, with polymerases either converging (green) or diverging (pink). (**b**) LOESS fit of the distance dependent correlation coefficients among sense, convergent, and divergent TSSs. (**c**) Comparison of the co-expression between neighboring and interlacing tTRE pairs to test the direct neighborhood interaction over distance effect (left). LOESS fits of the correlation coefficients in neighboring and interlacing tTRE pairs (right two panels). Note that neighboring pairs have higher correlation than interlacing pairs.

At close distances, RNA polymerases at one tTRE can potentially affect the neighboring tTRE in either a cooperative or inhibitory manner (Fig 4a). Recent works have suggested that convergent transcription near promoters and intragenic enhancers attenuate transcription from the gene through polymerase interference^23,29^. Others have shown that transcription read-through leads to increased chromatin accessibility^30–33^ and, therefore, cooperativity. It is also possible that transcription at one site may increase the local concentration of RNA polymerase and TFs, leading to rapid recycling of polymerase from one tTRE to the next.

**Figure 5:**
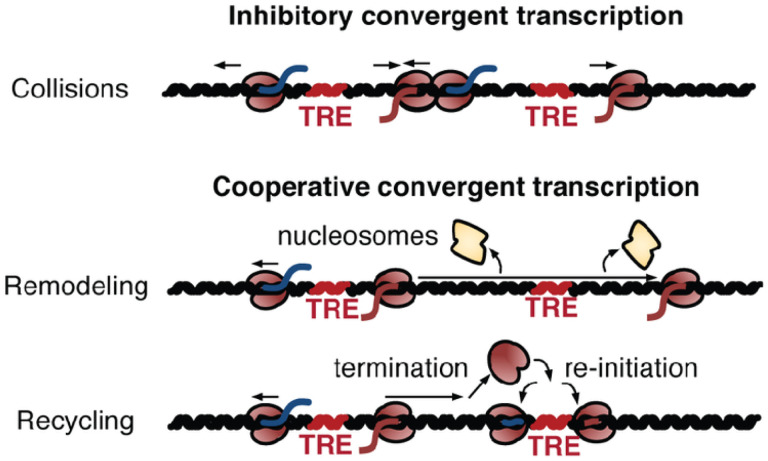
Covariation analysis supports a cooperative rather than inhibitory model for convergent transcription. Diagram suggesting three models of the transcriptional consequences of convergent transcription – collision, remodeling, recycling. Two adjacent enhancers (TREs) are engaged with bidirectional divergent RNA polymerase II (coffee bean shaped filled with red color) transcribing sense (red lines) and antisense (blue lines) nascent RNAs (see Discussion).

The strand-specificity of PRO-cap allows us to determine which model is more prevalent. If convergent transcription were inhibitory, the converging strands of two adjacent tTREs (upstream plus and downstream minus) would show lower overall correlation than the diverging strands (upstream minus and downstream plus) (Fig. 4a). We performed a strand-specific regression for all adjacent tTRE pairs and compared the distribution of the orientation-specific correlation coefficients. For closely-spaced tTREs (250 bp - 1kb apart), the divergent strand pairs are significantly less correlated than the convergent or the same strand (sense) pairs, even when the difference in the relative distance between the strands are corrected (Fig. 4b, **Supplementary Figs. 3a-c**). This pattern is no longer evident for tTREs that are far enough apart to make direct read-through unlikely (> 5kb) (**Supplementary Fig. 3c**). Also, if the enhancer pairs are interlaced by another enhancer in between, the convergent correlation pattern disappears (Fig 4c). These findings are more consistent with the model that convergent transcription is cooperative than inhibitory (Fig. 5).

## Discussion

We explored the activity, architecture, and functional interactions of eRNA transcribing tTREs using the variation in transcription initiation across human LCLs. The results reveal the principles of functional interactions between tTREs dependent on distance, TF binding, and direction of transcription.

Identification of transcriptional regulatory elements (TRE) based on capped nascent RNA sequencing provides a direct measure of transcriptional activity and, therefore, shows higher sensitivity. A direct measure of transcriptional activity is important, as non-productive transcripts such as eRNAs and uaRNAs are rapidly degraded in the nucleus. Other transcription-based approaches, such as CAGE and nuclear short RNA analysis, are impeded by this instability. As we showed, CAGE performs well in identifying promoters but less efficiently detects enhancers than PRO-cap. Additionally, by focusing on bidirectional eRNA transcription start sites (TSS) from PRO-cap data enabled us to filter out spurious transcription from only one strand. Taken together, we propose that PRO-cap provides a high quality set of tTREs.

Bidirectional transcription has been widely accepted as a general architecture of TREs. Our previous analysis of transcription initiation (tiQTL) and directional initiation quantitative trait loci (diQTL) implies that both the central TF binding and the core RNA polymerase initiating regions must be taken into consideration when assessing the potential impact of non-coding genetic variants^17^. Our model suggests a single TF binding site between the eRNA TSSs as a universal architecture, whereas other models have proposed two TF binding sites for promoter TREs, one for each strand^34^. Our interpretation is that two adjacent highly-directional tTREs form a larger nucleosome free region, where convergent strands are below the detection limit, and identified as a single TRE.

Beyond understanding individual TREs, we explored how TREs interact with one another to form cis-regulatory networks. Physical interaction maps have been the standard for identifying direct interactions. However, co-expression network approaches to identify functional interactions, though indirect, provide independent evidence for interactions and a good complement to physical maps. We were able to capture mid-range interactions (within 200 kb), which were dependent on distance and insulator/co-activator binding in a manner consistent with current knowledge. Interestingly, factors associated with strong promoters were insulating, indicating that strong promoters may dominate functional interactions and repress looping across them. This may be analogous to chromosomal boundaries in *Drosophila* that are formed by paused RNA polymerases^35^.

While most mid- to long-range interactions take place through DNA looping, the correlations we observed between close-range clustered tTREs also suggest a transcription-dependent mechanism (Fig. 5). Previous evidence indicated that polymerase collision leads to transcription termination between highly expressed intragenic enhancers and their host genes^23^(Fig. 5, top). However, most tTREs are less active, making simultaneous transcription and polymerase collisions rare. Instead, our strand-specific co-expression analysis suggests cooperativity rather than inhibition between convergent transcription in clustered tTREs (Fig. 5, middle/bottom). Perhaps transcription from a tTRE remodels the chromatin architecture in the neighboring region to increase accessibility (Fig. 5, middle). Another possibility is direct recycling of polymerase through termination and re-initiation at a neighboring tTRE, which could further be tested with a large-scale transcription-termination analysis (Fig. 5, bottom).

The identification of transcribed TREs using capped-nascent-RNA sequencing will be a powerful resource to the study of gene regulation. Although the identification of individual cis-regulatory networks may be limited due to the scale of our study, the principles and insights learned herein will help in predicting the regulatory targets of TREs and understanding their biological consequences.

## Methods

### Co-expression analysis of tTREs.

We used the variably expressed tTREs (n=29,694; promoter - 4,006; enhancer - 25,688 using the CAGE based criteria), and calculated correlation coefficients of the 75 individual normalized readcounts (67 individuals + 8 replicates) between two tTREs within 5 Mb distance (2,249,839 pairs). For the distance analysis, we binned the correlation coefficients by the distance between 2 tTREs. The bins are generated based on fixed distance intervals up to 1,024 kb, or a fixed number of tTRE pairs (1,000 pairs per bin) with variable distance intervals. With the variable interval bins, we generated plots for the 5th percentile, median and the 95th percentiles of the correlation coefficient distributions within each bin of 1000 pairs along the distance (**Supplementary Figure 1a, 1b**). To compare the tTRE correlations independent of the genotype correlations in the population structure (linkage disequilibrium), we used genotypes at the SNPs nearest to the TSSs with the minor allele frequency greater than 0.05, and selected tTRE pairs within 1 Mb distance whose associated genotypes are not correlated (|correlation coefficient of the genotypes| < 0.05, n=129,660 pairs; **Supplementary Figure 1f**).

### Co-expression analysis between tTRE nascent transcription and mRNA expression.

We used the RNA-seq expression data from Pickrell et al.^36^ and selected 13,002 genes with the mean expression level greater than 1 RPKM. 275,660 pairs of tTREs and annotated mRNA TSS within 1 Mb were tested, and the correlation coefficients of the 67 individual samples were calculated.

### Analysis of the ENCODE factor dependencies in tTRE co-expression.

The top 5 percentile of the correlation coefficients in the variable interval bins was used as an indicator of tTRE co-expression. This correlation coefficient percentile serves as a function of the distance, and we refer to it as the correlation decay plot. To test whether the abundance of ENCODE FACTORBOOK binding sites - located either between the tTREs or at the tTREs - affect the correlation decay plots, we calculated the difference between the area under the correlation decay curves (ΔAUC) in the 0 – 200 kb distance range. The significance of the ΔAUC was estimated by comparing to the background distribution of ΔAUC which was generated by random shuffling of the factor binding sites. We calculated the background ΔAUC distributions by 100 permutations of 5,000 – 70,000 randomly shuffled ENCODE factor binding sites in GM12878. The background ΔAUC distributions followed a normal distribution and were dependent on the number of binding sites. We approximated the expected mean and standard deviation (sd) of the ΔAUC dependent on the actual number of the specific factor’s binding sites, and used it to generate a z-score and p-value of the ΔAUC between correlation decay curves enriched or depleted with the factor binding sites. For example, CTCF contains 41,465 ChIP-seq binding sites^27^, and the ΔAUC = +27.8 between more than 2 CTCF sites or less than 1 site intersecting a pair of tTREs. The background distribution of ΔAUC with 40,000 sites intersecting tTREs is +6.41 ± 2.56 (mean ± sd), and we obtained the z-score = +8.34 and p = 7.26 × 10^−^17 for CTCF intersections affecting correlation decay plots. The intersection and the occupancy scores of all other 76 factors available for GM12878 in FACTORBOOK were calculated in this way. Only factors with sufficient number of binding sites in each category were reported. These p-values and z-scores were used in hierarchical clustering and t-stochastic neighbor embedding (tSNE) analysis to cluster TFs.

### Strand-specific co-expression analysis for tTRE nascent transcription at adjacent sites

For each tTRE, the nearest neighboring tTRE downstream was identified and a linear regression between their strands was computed across the samples according to the following categories: Sense (plus-plus, minus-minus), antisense convergent (plus-minus), and antisense divergent (minus-plus). Pairs of tTREs were binned based on the distance between them and the distributions of Pearson correlation coefficients were compared between the categories. Pairs within 250 bp were not included to avoid any possibility of the same read being counted in both tTREs.

## Data Availability

The data can be accessed through the GEO (GSE110638). The datasets generated and analyzed during the current study are available in the Github repository, [https://github.com/sl2665/procap-network].
